# Supplementary material for: A predictive model of the effect of therapeutic radiation on the human ovary
Source: PLoS One. 2022 Nov 18;17(11):e0277052. doi: 10.1371/journal.pone.0277052 (PMC9674157; doi:10.1371/journal.pone.0277052)
Supplement: Attachment — Submitted filename: ResponseToReviewers.docx [file pone.0277052.s001.docx]

Rebutttal letter PLoS ONE

PONE-D-22-15540

A predictive model of the effect of therapeutic radiation on the human ovary

PLOS ONE

Dear Meijia Zhang Academic Editor PLOS ONE,

Thank you for your detailed and helpful reviews of our manuscript.

1. Please ensure that your manuscript meets PLOS ONE's style requirements, including those for file naming.

*We have carefully considered PLOS ONE’S style requirements and made any changes that were necessary. See revised manuscript.*

2. In your Data Availability statement, you have not specified where the minimal data set underlying the results described in your manuscript can be found. PLOS defines a study's minimal data set as the underlying data used to reach the conclusions drawn in the manuscript and any additional data required to replicate the reported study findings in their entirety. All PLOS journals require that the minimal data set be made fully available. Upon re-submitting your revised manuscript, please upload your study’s minimal underlying data set as either Supporting Information files or to a stable, public repository and include the relevant URLs, DOIs, or accession numbers within your revised cover letter.

*The minimal data set underlying our results is the solution to the Wallace-Kelsey model which is publicly available in Table form in the java script directory of the web page. We have added a data availability statement in the manuscript specifically so that the reader can if required source the original data set.*

Reviewer 1 and 2 specific comments:

1. Is the manuscript technically sound, and do the data support the conclusions?

Reviewer #1: Yes

Reviewer #2: Yes

*Thank you for these positive assessments.*

2. Has the statistical analysis been performed appropriately and rigorously?

Reviewer #1: Yes

Reviewer #2: Yes

*Thank you for these positive assessments.*

3. Have the authors made all data underlying the findings in their manuscript fully available?  The [PLOS Data policy](http://www.plosone.org/static/policies.action#sharing) requires authors to make all data underlying the findings described in their manuscript fully available without restriction, with rare exception (please refer to the Data Availability Statement in the manuscript PDF file). The data should be provided as part of the manuscript or its supporting information, or deposited to a public repository. For example, in addition to summary statistics, the data points behind means, medians and variance measures should be available. If there are restrictions on publicly sharing data—e.g. participant privacy or use of data from a third party—those must be specified.

Reviewer #1: Yes

Reviewer #2: Yes

*Thank you for these positive assessments. See our response above regarding public availability of the data set.*

4. Is the manuscript presented in an intelligible fashion and written in standard English?  PLOS ONE does not copyedit accepted manuscripts, so the language in submitted articles must be clear, correct, and unambiguous. Any typographical or grammatical errors should be corrected at revision, so please note any specific errors here.

Reviewer #1: Yes

Reviewer #2: Yes

*Thank you for these positive assessments.*

5. Review Comments to the Author

Reviewer #1: 1.The authors should give a specific definition of the non-growing follicle(NGF).

*An accepted definition of the non-growing human follicle is as follows: Non growing follicles (NGF) contain immature oocytes surrounded by flat, squamous granulosa cells (support cells) that are segregated from the oocyte's environment by the basal lamina. They are quiescent, showing little to no biological activity. They represent the human ovarian reserve. We have inserted this definition into the introduction and standardised our terminology throughout the manuscript using NGF throughout.*

2.Line 172 and 173: the reference was outdated.

*Thank you. We have updated the reference in relation to the assessment of ovarian reserve in those women under 25 years to PMID 32315387, published in 2020.*

3.Figure 4a: the line chart of the proton plan lacked a Y-axis title.

*Thank you. We have corrected this error. The y-axis is % coverage as in the other figures.*

Reviewer #2: The POI caused by radiotherapy for cancer in young women has a significant impact on women's fertility. This study provides a model for predicting the age of occurrence of POI and allows detailed predictions of the impact of various radiotherapy plans on fertility, which helps to understand the fertility risk of patients more clearly.

*Thank you for these positive comments.*

1. The four cases in this study are fewer, the coverage should be more comprehensive.

*The four cases are exemplars. The aim of the four cases is to demonstrate the potential application of using our algorithm to predict with confidence intervals the age at POI. This is useful for counselling and for discussions around fertility preservation. We think more cases will not add anything to the manuscript as they illustrate the methodology in four representative patients and are not the results of our manuscript.*

2 There seems to be a problem in the description of the method, for example, lines 134-146 seem to be in the result.

*This comment refers to our methodology for the models that underpin the algorithm as shown in figures 2 and 3. We are happy to accommodate this referee’s comment and have transferred the section that describes Figures 2 and 3 (lines 134-146) to the results section.*

3 It is confused that the description of fig.1, fig.2 and fig.3 appear in methods.

*We have, as above, transferred the description of figures 2 and 3 from the methods to the results section.*

4 The results of fig.4-fig.7 are too scattered and should be integrated. Why are the figures separate from each other in fig.4abcd? Also include fig.5 abcd, fig. 6abcd, fig.7abcd?

*Each of the figures 4 through figure 7 represent patient exemplars with each example having four sub figures. We do not disagree with the sentiment of the reviewer, but if they are combined into a single figure with four panels we cannot retain the fine detail and retain image publication quality required by your journal.*

5 The structure of this manuscript is a little confused

*We hope that by moving the explanation describing figures 2 and 3 into the results section the structure of the manuscript is easier to follow.*

We sincerely hope that the revised manuscript is now acceptable for publication in PLOS ONE

Yours sincerely

Prof Tom Kelsey
